# Intersectionality, equity-mindedness, and access to care

**DOI:** 10.3389/fvets.2025.1602950

**Published:** 2025-07-24

**Authors:** Naomi W. Nishi, Erin Watley, Mary Jane Collier, Gabriela I. Morales

**Affiliations:** ^1^College of Veterinary Medicine and Biomedical Sciences, Department of Biomedical Sciences, Colorado State University, Fort Collins, CO, United States; ^2^Department of Communication and Cinema, McDaniel College, Westminster, MD, United States; ^3^College of Veterinary Medicine and Biomedical Sciences, Department of Clinical Sciences, Colorado State University, Fort Collins, CO, United States; ^4^Department of Communication Studies, New Mexico State University, Las Cruces, NM, United States

**Keywords:** veterinary care, intersectionality, equity-mindedness, access to care, race, gender, intersectional equity-mindedness

## Abstract

The factors that contribute to an individual’s and/or community’s access to veterinary care for their animal(s) are manifold. While some (potential) clients must navigate barriers, including transportation options, time off during business hours, physical access, cost of care, and language barriers, there are also identity factors and experiences that affect access to veterinary care particularly for people whose identities are marginalized. These include clients experiencing distrust and/or disrespect in their interactions with veterinary professionals. We explore how intersectionality affects access to veterinary care, especially for those who are multiply marginalized by societal systems of oppression. We share research findings on the experiences of clients that identify as women of color and/or non-binary people of color that have had to navigate barriers to their access to veterinary care, including through broken trust and experienced disrespect. We then define and propose strategies for engaging intersectional equity-mindedness in clinical practice. We connect these concepts of intersectionality and equity-mindedness to their implications for access to care.

## 1 Introduction

We, the authors, would be remiss not to acknowledge the sociopolitical climate in which we have conducted this research and in which we have prepared this manuscript. While our interviews were conducted in 2021–2022 near the ‘end’ of the COVID pandemic, we have prepared this manuscript to highlight the importance of considering the intersections of gender and race in veterinary medicine at a time when this very topic and the words being used to describe it are under attack. Within this attack, language and words such as diversity, equity, and inclusion are being misused and co-opted in such a way to suggest that they are discriminatory when the work they describe has been steadfast in its combatting of racial, gender, and other forms of discrimination deeply embedded in the US national fabric. We refute this co-option and misuse and offer this work as justification for the importance of not only continuing but also growing social and health justice to expand access to veterinary medicine and care.

The field of veterinary medicine, and particularly companion/pet animal care, has a history of racial exclusion, both as a profession (93.8% of veterinarians are White) (1) and as a service. Wolf et al. (2) found that people of color were not as likely to use veterinary services compared to their White counterparts and called for development of diversity and cultural competence within the profession as a response. In more recent research, King et al. (3) found that race and ethnicity also impacted pet owners’ perceived access to veterinary care^1^ (4). Whereas traditionally, pet ownership has been disproportionately White, families of color continue to increase their rates of having at least one companion animal. Approximately 70% of White people have a pet, and 29% of African Americans and 60% of Latinx people have one or more pets (5).

Beyond race, US veterinarians and Doctorate of Veterinary Medicine (DVM) students do not reflect the diversity of the US population, particularly when it comes to marginalized and minoritized identities. Most veterinarians and DVM students come from middle-and upper-class backgrounds (6). Most identify as able-bodied and/or neurotypical. A disproportionately small group of DVM students identify as LGBTIQA+ (6). This lack of diversity can lead to some people from these marginalized communities not seeing themselves reflected in the professional veterinary workforce, which has pipeline implications, hearkening the saying, ‘if you cannot see it, you cannot be it’ (7), but this lack of diversity also leads to the reinforcement of those with dominant identities being the standard (and being centered) along with their ways of understanding and operating in the world and the profession.

This creates clinical environments where those with marginalized identities, both providers and clients, not only feel like, and often are, ‘the only one,’ but also have to combat the negative stereotypes and microaggressions without any support or persons to relate to or validate those experiences (8). Relatedly, Park et al. (9) found that dog owners from some communities of color (Black/African American and Native American) noted that a lack of trust in veterinarians was a barrier to their access to care. This is all in an environment where pet ownership among people of color is increasing (10) but communities of color and those with low socioeconomic status perceive a lack of access to veterinary services (3).

It is worth delving into a definition of trust, which we invoke as a foundational concept in this work. While this concept has been explored within veterinary medicine, it is not always well-delineated. Some have connected it to perceived competence (11). Similarly, ability, integrity, benevolence, vulnerability, and risk taking are all related to trust or are overlapping the concept in a Venn diagram (12). These then beg the question of what goes into a client ‘trusting’ their veterinary care provider and their ability? In addition, how does trust allow for the belief by the client that their veterinarian has their and their animal’s best interest at heart?

Understanding an animal’s history from a client is most certainly an essential component to that animal’s care. So, how might trust be broken and care potentially compromised when a client does not feel heard or thinks their veterinarian is making (false) assumptions about them/their animal? Relatedly, if we think about trust built in relationship, when we think a veterinarian mistrusts, or thinks less of us, how does this then reciprocate in our own feelings of mistrust or thinking less of the veterinarian?

In this interview-based study, we sought to answer the question, *what are the experiences of women and nonbinary people of color in veterinary settings?* for those who are clients and those who are veterinary healthcare providers. In what follows, we describe intersectionality and equity-mindedness and share how these phenomena unveil barriers to veterinary care, particularly for clients who identify as women or non-binary people of color. We share the results from our client participants and offer how intersectionality and equity-mindedness can provide a lens and tools to increase access to care for these communities. While our research project included participants who were clients and those who are veterinary professionals, the scope of this article includes perspectives only from client participants. We explore veterinary professional experiences in forthcoming articles.

## 2 Theoretical framework

Intersectionality is a term coined by critical legal scholar, Kimberlé Crenshaw, in her explication of the ways that Black women are not served and rendered invisible by the legal system. This is because their experiences are situated at the intersection of White Supremacy and Patriarchy (13). Drawing on Crenshaw and other Black Feminist scholars’ work (14–16) in veterinary medicine, Nishi et al. define intersectionality as “a concept that holds identities and systems of oppression as interrelated and connected. In this framework, the intersectionality of systems multiply^2^ marginalizes those with compounding marginalized identities, such as people of color, women, trans and/or non-binary, and people from low socioeconomic status backgrounds” (8).

Crenshaw (13) cites the legal case, *DeGraffenreid v. General Motors (1976)* (17), as the grounding case for her conceptualization of intersectionality. Emma DeGraffenreid was a Black woman who sued General Motors for hiring discrimination because she was a Black woman. The suit was thrown out in court because General Motors showed that they did hire Black people (they had Black men working on their factory floors), and they did hire women (they had White women working in clerical roles). The court refused to see that it was DeGraffenreid’s intersectional identities of Black *and* woman that led to her discrimination. This case and others like it, brought by Black women, exposed to critical legal scholars and critical race theorists that the legal system, created by and for elite White men, rendered those with multiple marginalized identities invisible. The legal system did not consider or account for Black women, and so they were the first to fall through the cracks.

Other Black, feminist scholars have developed the foundations of intersectionality. Patricia Hill Collins (14) described “the matrix of oppression,” demonstrating how oppressive systems such as racism, cisheterosexism, ableism, and classism work together to “multiply marginalize” some and hyperprivilege (18) others. This matrix and the interconnected nature of these reinforcing systems of oppression hold exponential consequences and harm for those at the juncture of multiple systems.

Research in human medicine offers us insight into how we can understand the ways that intersectionality works within the lived experiences of those navigating the world of veterinary medicine. In what follows, we identify some of the health disparities encountered by communities with marginalized identities and then look at the intersectional experiences by those with multiple marginalized identities.

Communities of color, and particularly Black, Latiné, and Native Americans, experience vast health disparities, including from subpar healthcare they are provided. This includes undertreatment of pain for Black people (19, 20). Looking at gender, we see women experiencing disparities as they are less likely to be believed and/or their concerns written off as emotional by their care providers, including related to belief around women’s pain (21). LGBTIQA+ people experience health consequences for a health system designed for cisheterosexual patients (22). Of course, poor and working-class people who are uninsured or underinsured often forego preventative care and only seek care in emergencies when the health and financial consequences are much higher (23–25). Related to ability, Lagu et al. found that providers tended to assess the quality of life of those in wheelchairs as lower than able-bodied patients and in some cases did not find a way to have wheelchair bound patients get out of their chairs for exams (26).

From these examples of the health disparities wrought by the human healthcare system for people with marginalized identities, we can see how people who hold multiple marginalized identities experience exacerbated consequences. For example, we see extraordinarily high rates of maternal mortality for women of color and particularly Black women (27). In addition, women of color report elevated levels of medical gaslighting, where they are ignored, doubted, and second guessed by healthcare providers to such a level that they begin to doubt themselves and their own experiences (28).

Although this research is in human healthcare, we can see the possible implications that these human-as-patient experiences can have for human-as-client in veterinary medicine. For example, women might not be believed or be written off as emotional when describing their animal’s symptoms rather than their own. Some clients of color might be assumed to not be able to afford their animal’s care (8). Providers might presume clients who appear to be from poor or working-class communities do not care for their animals as much as wealthier-presenting clients.

*Equity-Mindedness* is a concept coined by Estela Bensimon (29, 30) and developed within higher education (31–33). Equity-mindedness is the application of a critically conscious schema that highlights racial disparities in an organization and engages the organization’s agents in remedying and re-designing processes, policies, and initiatives to combat racial inequity (32–35). It is inherently a critical and anti-deficit framework focused on racially minoritized community members (29, 30, 36).

Bensimon and Malcolm advocate for reframing ‘the problem’ and the responsibility for racial inequity as that of the organization, and its programs, policies, culture, and mindset, rather than problematizing people of color within the organization (34). The work then shifts from that of improving or ‘helping’ marginalized people to removal of the barriers for people of color through dismantling and re-developing of systems that are racially inequitable, as well as through educating the larger organization on the issues of inequity.

Equity-mindedness has largely been developed within education, and particularly higher education. Yet, the systematic approach to reframing racial inequity is applicable to other organizational types and professions. In the way that one can use equity-mindedness to analyze racial disparities in education, so too can we use this framework to analyze inequity in healthcare, both human and animal. As the field of veterinary medicine evolves to more comprehensively consider the human social impacts in the field, we are advocating for transdisciplinary approaches from the social sciences, including communications studies and critical education research, to promote the adoption of equity-mindedness.

Bensimon and others have unabashedly focused equity-mindedness on *racial* equity (29, 30, 32–34). Bensimon warns against the drift away from a lens trained on race, describing it as a ‘lethal mutation’ (30, 37) when we shift to focus on other equity issues. She argues that this drift can result in ignoring or overlooking the ways that racism works within our systems. Instead, many choose a more palatable approach (e.g., focusing on first generation or socioeconomic status), where people and organizations are able to avoid considering their complicity with racism. Unfortunately, this lethal mutation is all too common in research and practices that apply a critical lens to race, whiteness, and white supremacism. Multiple scholars have described the cooption and redefining of concepts within Affirmative Action legal precedent and diversity policy in higher education that water down a racial lens, rendering it inactive, or reframing it to focus on the educational benefits of diversity for White students (38–40). Harper (41) demonstrates a preponderance of higher education research that refers to race without any mention of, let alone meaningful engagement of racism. These same analyses and lessons learned can be easily applied within healthcare and veterinary medicine. For instance, we also see a preponderance of literature in healthcare that notes race, but very rarely do we see that same body of literature invoke *racism*.

Yet, with acknowledgment of the dangers of watering down or ‘whitening’ the concept of equity-mindedness, we suggest that there is also a danger of staying so laser-focused on race that those who are marginalized by race *and* by other systems of oppression (e.g., Patriarchy), will fall through the cracks of solutions and remedies to policies, practices, and climates that are only oriented to race as *Intersectionality* has demonstrated. Thus, our theoretical framework employs a hybrid of these concepts that we refer to as *Intersectional Equity-Mindedness*. We suggest that this concept is intersectionality operationalized, where our analysis centers those who are multiply-marginalized.

## 3 Materials and methods

Research in veterinary medicine tends to use quantitative methods, and because our study is qualitative, we wanted to offer some framing for clarity around some common misconceptions around qualitative research. First, qualitative methods are designed to understand and/or make sense of phenomena, rather than to generalize through statistical significance, which is often the goal of quantitative research (42). While quantitative researchers are concerned with sample size, qualitative researchers focus on saturation of concepts in their data and yielding thick descriptions (43) to vividly explain their focus (44). While quantitative researchers look at frequency, qualitative researchers look at resonance (how themes emerge in different ways from the data) (45).

The question of reproducibility is quite rightly being raised and demanded across academia and particularly connected to medical research. However, when we hear this concept, we tend to think of it from a quantitative and experimental lens. When we are engaging in qualitative research, we can and should expect that the themes emerging from the data will be transferable in other contexts and other qualitative studies. But, given that this research focuses on people’s experiences and their narratives, it is inappropriate to apply an experimental/quantitative standard of reproducibility to most qualitative research (46, 47). Thus, in what follows, we do not seek to prove that women/non-binary people of color have experiences of intersectionality in clinical settings. Rather, we seek to illustrate some ways that women and non-binary people of color experience intersectionality in veterinary clinical settings.

As a critically reflexive move, our research team’s cultural identifications, professional positions, and areas of research praxis are important to clarify for the reader. Our research team consists of four university faculty members who identify as Black American, Mexican American, and White cisgender women. Our cultural, group, and professional identity labels are self-selected. Labels for identity groups should be self-selected, and meanings clarified for readers because labels signify inclusion, exclusion, and can become shorthand for stereotypes. We study cultural diversity and communication and culture along with systemic oppression and marginalization in health, academic, public discourse, and veterinary settings. We hold appointments at three different academic institutions.

In our overall program of research, we sought to outline first-hand experiences described by clients and veterinary practitioners related to race, gender, and other cultural identities. In 2021 and 2022, we interviewed clients and veterinary professionals who identify as women or non-binary persons of color.

In this study, we focus on a theme we identified early on in our coding, the harmful outcomes clients described resulting from their first-hand experiences of overlapping/intersectional racism, sexism, classism, ageism, nationalism, and regionalism during their clinical appointments. Please note that we address the responses of the veterinary professionals compared with client responses in a forthcoming article. For this research project, we obtained Institutional Review Board (IRB) approval at three different academic institutions.

We recruited clients through a particular social media discussion group of US academics at all levels from diverse disciplines. The group has some 10,000 members who identify as persons of color (POC); as women or non-binary, and have an interest in encouraging, mentoring, and supporting women and non-binary people of color in academia. The group is private and individuals must be approved to gain membership. Two of the members of our research team are members of this group. They posted an announcement recruiting participants to be interviewed for our study.

The recruitment text posted on the social media site described that to be eligible to participate in our study, clients had to be individuals who are at least 19 years old, currently have or have had a companion animal that they have taken to a veterinarian for care, identify as a woman or non-binary person of color, and be able to describe their experience of communication as a client with a veterinarian that was impacted by race and gender, among other cultural identifications.

Participants filled out a demographic form as well as signed informed consent per IRB guidelines. To protect confidentiality, each interviewee filled in their own labels for race, ethnicity, sex/gender, and any other cultural identities that were salient for the client interactions with the veterinary professionals. The Race/Ethnicity (Table 1) and Sex/Gender (Table 2) demographic tables list participants’ preferred labels for their group identities. These self-generated labels are important because one’s gender can be non-binary and fall on a wide spectrum of identities. Similarly, while racial/ethnic groups such as ‘Asian’ are commonly used to stereotype or position others, individuals may avow (self-identify) with more particularized groups such as Japanese American or Chinese American, and individuals can be mixed race. Since our focus was on eliciting individual experiences related to cultural identities, using an inductive, open-coding approach (48), cultural identity is not a discrete demographic variable where individuals are asked to “check a box” and researchers predict or explain conduct on that basis.

**Table 1:** Participant racial/ethnic identities.

Identity	Clients	Veterinary professionals
Hispanic	1	1
Latina	3	1
Latiné/x	4	1
Peruvian American/Latina	1	
Mexican/Latina	1	
Black	3	1
African American	1	2
Black/African American	2	4
Asian	1	
Asian/Chinese American		1
Asian/Chinese-Filipino	1	
Japanese American		1
Mixed race: Hispanic/White; Indigenous/Hispanic	2	
Mixed race: Black bi-racial; Asian American	2	
Mixed race: Afro-Latina, African American, Native American		3

**Table 2:** Participant sex, sexual, and gender identities.

Identity	Clients	Veterinary professionals
Female/Woman	12	14
Cis-woman, cis-female	2	1
Female bisexual	2	
Non-binary	3	
Female LBTQ	2	
Queer	1	

Participants also offered their own pseudonyms for themselves and the veterinary professional with whom they interacted. For each response quoted in the body of the study, we list the pseudonym and whatever combination of race, ethnicity, nationality, sex, sexuality, gender, and professional identity group label each interviewee listed on the demographic form or described as salient to their recalled interaction in the veterinary setting in the interview. For our overall program of research, 12 of the women/non-binary people of color respondents are veterinarians, 3 are licensed veterinary technicians, and 22 are veterinary clients.

The interview guide was developed in meetings among the research team. We first reviewed our overall goals of examining first-hand accounts of women/non-binary persons of color describing their experiences with veterinarians. We also agreed that we wanted to better understand clients’ views of the outcomes from such interactions as well as the harms and/or benefits produced. Second, we reviewed our theoretical perspective, which is intersectionality and uncovering how the clients experience being positioned, and perhaps stereotyped on the basis of their cultural identities, such as those based on race and gender, as well as other group identity positions such as class, nationality, or generation. Given our methodological goals of having the participants describe what they experienced as significant, as well as the outcomes from the interactions, we then created general open-ended questions to elicit clients’ narratives about their communication with the veterinarians and their staff. These questions began with, “Please talk about your experience with a veterinarian and how race and gender were relevant.”

Our interview guide consisted of broad questions. Most of the clients were interviewed in focus groups of two or three clients. They were recorded on Zoom. For the clients, we asked them to recall an appointment with a veterinarian and then to share how race, sex, gender, and other cultural identities impacted the communication. Probes for the clients included questions such as, “What did each person say and do? What did you say and do? What were outcomes from the interaction? To what extent were you able to develop a partnership relationship with the veterinarian?” After each individual had shared, the group was asked to offer suggestions (for the veterinary professionals and themselves as clients) of how to make the communication more effective. They were also invited to share any other information they wanted to add.

Three faculty members conducted the interviews. The two research team members who also identified as women of color conducted the interviews with clients. This move was to engage their lived experience and knowledge of the complicated political and social context in the US around race and gender. In addition, their publications and teaching are in intercultural communication. The research team member who conducted the interviews with the veterinary professionals has taught and coached veterinary students for several years in a DVM program, as well as co-facilitated workshops with residents, interns and with practicing veterinarians and veterinary technicians, and has published a body of work on cultural identities such as race, gender, nationality, and class. Her familiarity with the veterinary context, as well as the challenges faced by people of color in the profession, helped build trust with the interviewees.

We conducted interviews because citing examples from individuals’ actual encounters increases the validity of our interpretations, shows the resonance of the experiences, demonstrates that processes like racism occur in different places and spaces, and increases the relevance of suggestions offered (49–51). To further increase validity and protect confidentiality, participants were contacted for a Member Check of interview transcripts by the interviewer and asked to approve/edit their interview comments, cultural identity labels, and description of their professional positions.

Our coding procedures involved several systematic steps. Building on Owens’ thematic analysis of interpersonal communication (52), we endorse Denzin’s call to adopt “a humanistic and social justice commitment to study the social world from the perspective of the interacting individual” (53). In addition, we apply the work of Lawless and Chen as they outline an approach to coding interview texts for themes from interviews that reflect social systems that produce harm such as racism, sexism, classism, and nationalism (48).

More specifically, as an initial step, all the researchers read the interview transcripts to get a holistic sense of the responses, as Denzin advises (53). In the second step, the three interviewers coded their own interviews to identify themes for two reasons. Personal experience with the contested context around race and gender in the US and knowledge from member checks, or personal experience engaging the veterinary context and member checks were important to increase validity. Second, each of us specializes in applying a critical lens to communication texts that include narratives to address how racism, sexism, and classism work; this was the first step of our coding process.

Again, our study was designed as inductive, open-coding, uncovering any patterns in client and veterinary professional experiences in clinical settings, not to test or generalize about variables from past research; the third step was for each member of the research team to suggest common themes in types of messages seen and heard, and outcomes experienced in their interview transcripts (48, 53). Then, a written draft of the messages was shared with other members of the team. The fourth step was to have Zoom meetings to discuss preliminary observations with the rest of the team. We each noticed that narratives included harms experienced from overlapping/intersectional racism, sexism, classism, ageism, nationalism, and regionalism, and the prevalence of microaggressions (8) from both clients and veterinary professionals.

Then, each interviewer organized their draft of direct quotations of examples of intersectional racism, sexism and so on, and of microaggressions, from their clients or veterinary professionals interviewed, and shared them with the rest of the team. Pertaining to the client experiences here, we included quotations about what these messages and interactions produced in the way of outcomes such as mistrust in the care options offered or feeling disrespected. Then, we had several more Zoom meetings to discuss which quotations were the best examples to illustrate what the clients were experiencing.

Related to reliability, all members of the team agreed upon the examples cited and how they were categorized into themes or codes, such as intersectional racism and sexism, or racism and classism, and so on, and/or a microaggression (8). Thus, reliabilities are based on consensus which emerged through dialogue. See Collier for an application of such a coding approach (54). We selected the best examples to include in published manuscripts, rather than producing any sort of frequency count.

Then for this study, to address readers’ questions for “What do we do with this information on intersectional racism and sexism?,” we also discussed actions that veterinary professionals could take to pre-empt the harmful communication. One member of our research team has extensive experience teaching medical and veterinary professionals about social and health justice, particularly related to racism and sexism, another has experience teaching veterinary communication in a DVM program related to building communication skills systematically and dealing with case scenarios with diverse clients; and all four researchers have extensive experience teaching and researching critical intercultural communication, including issues around race, sex, gender, class, and other cultural identities. What appears below are examples, outcomes, and suggestions on which the team reached consensus about the type of intersectionality evident, the outcomes experienced by clients, and preliminary recommendations for DVM professionals and programs to prevent and manage the harmful communication.

We want to recognize the limitations of our study. All of our participants reside in the United States, and thus, the cultural contexts are similar in some ways. Although our research team is diverse, we too all reside in the United States and are all faculty members in US higher education institutions, which might limit our perspectives in some ways. Certainly, all members of the research team have our own experiences, contexts, and biases that impact our coding and interpretations and sense-making processes, but, although these limit us in some ways, they also strengthen our analytical lenses as we work together to illustrate the phenomenon of intersectionality. Finally, another potential limitation could be the sourcing of our participants through academic-affiliated social media in terms of potential similarities of bias and background of participants.

## 4 Results

From our focus groups and interview data emerged the theme of intersectionality, or how our participants’ marginalized identities, particularly by race and gender impacted their experiences in veterinary spaces. In particular, we noted the trends in our participants’ intersectional experiences around *White/men deference*, *White paternalistic disrespect*, and *racial/gendered gaslighting.*

As clients, many participants noted that even when they were the primary caretaker for their animal, they experienced the veterinary team deferring to White friends or family members, and particularly White men. One woman, Tyler, who identifies as mixed race, specifically Black and White, shared her experiences seeking veterinary care for her cat.

I feel like when I’m with my [White] husband at the vet and they are then suddenly more receptive to his question and answering his questions and it does not feel like they are trying to tell him what he has to do, or [what] we have to buy or questioning are we cleaning the litter box out? I get those questions like ‘Oh, are you cleaning the litter box out enough?’ when I go alone. Like this very parental type of condescending in a way. Then I go with him [husband], and they direct questions to him and I’m sitting there like I do a lot more for the cat but like it’s almost like he’s the cat parent. Then the questions are directed towards him, and the information is shared with him and I’m like ‘Hello!’ Then it causes me to shut down because I’m like ‘Okay, whatever.’ So, then I check out and he speaks with them and they laugh, and he has like a fine experience. He does not know what it’s like when he’s not there and I try and explain that it is just frustrating. It’s fine when I go with him and it just sucks to feel like I need him to be with me in places to allow my questions to be answered, or for it to be taken more seriously. When I go I [get ready to] leave and it’s like, ‘Here are all the pills they should take. It’s going to be estimated [specific] amount of dollars.’ When he’s there we do not leave with that huge thing [pills/bill?] and I’m just like is that a coincidence, or is that part of the genuine experience?

Tyler also connects her current experiences seeking veterinary care with those she had as a child, as the daughter of a Black mother and White father. She shares,

I’m thinking of my experiences as a kid too…I used to live up in the mountains and there it was like a really, really White area, and so the same thing they are going into the vet. My mom is my Black parent, she would go and take our dog in, and it was a very different experience than when I would go with my dad who is my White parent plus he, like knew the vet so it was all like Oh, you know, buddy-buddy. It was a male vet at that one, and it was always like so different than going with my mom.

As an adult, Tyler describes her experience when her White husband is with her and the veterinary care providers deferring the conversation and recommendations to the White man in the room even though Tyler is the primary caretaker. She also contrasts the difference in tone and conversation when she is at the veterinarian without her husband, describing the tone of the veterinarian as “parental” and “condescending.” Tyler clearly describes this theme of White paternalistic disrespect and notices this behavior because of her intersectional identities.

Tyler also connects this with her experience growing up, witnessing the differences in conversation between her White, man, veterinarian and her Black mother versus her White father. She describes the relationship between the vet and her Dad as “buddy-buddy.” This echoing of White paternalistic disrespect punctuates Tyler’s lifetime experience with veterinarians. Notably, the changing of the veterinarian’s gender, whether a White man or White woman, did not change her experience of White man deferral or White paternalistic disrespect.

Tyler describes the impact of this experience. She talks about experiencing frustration and then shutting down in an almost self-protective response. She disengages when she experiences disrespect and/or deferral to her White husband, but she later describes that frustration resurfaces along with self-doubt when trying to communicate with her White husband what she is experiencing. She notes that she wonders if it is just a coincidence that upon leaving she is offered different things (i.e., medication) in different ways depending on if she is there with her husband or alone. This illustrates the insidious effects of racial/gendered gaslighting and is reminiscent of the medical gaslighting implications (28), where Tyler feels disrespected and doubted by her veterinarian and then begins to self-doubt if what she is experiencing is all in her head. Tyler’s intersectional experience of racism and sexism in subtle ways results in her questioning herself, and her experience, and sustains her frustration and exhaustion even after she has left the veterinary clinic.

Other women of color noted similar experiences of particularly White deferral. Yolanda, who identifies as an Indigenous woman, describes this White deferral to a White woman, sharing,

What would often happen is that the veterinarian would just instead of talking to me, knowing that this is my cat, he would talk to her [brother’s girlfriend who is a White woman and would accompany on the visits]. And he would say ‘Well, this is what he needs. He’s doing really well.’ And all that conversation would be directed to her. Okay, well, I take care of them, so I do this, and so the veterinarian would then start talking to me, but then he’d again start to drift off and start talking to her, and so I would try to insert myself into the conversation. That has not been the first time that’s happened with a veterinarian and even my interactions with the vet techs we have gone to at one point. There was a low-income clinic where we went to get like just like flu shots or something and we took cats and all there was maybe like three vet techs and a veterinarian doing all this work, and it was the same thing. They would talk to her instead of me, and it did not feel great, and it was just the same pattern I kept seeing over and over again.

Yolanda describes that at various veterinary clinics and appointments, when she was there with her friend who was a White woman (not even one who was a family member), the providers, including the veterinarian and vet techs, would talk to the White woman. Yolanda even describes intervening and directly sharing with her provider that she was the cat’s caretaker and describing how she was caring for her cats. When she called this out, the providers would respond by momentarily re-directing the conversation to her only to inevitably return the direction of communication to the White woman in the room.

Lunankin, who identified as an Asian woman, described a similar experience of veterinary professionals deferring to the White man in the room as well.

Especially with my partner, it’s always kind of, because my partner is a U. S. White American man they all tend to kind of like talk to him, look at him, even though I am the one that is initiating and asking questions, even though, like, for example in the past few months, this year, when I take my, when I took my cat to the vet in Minnesota, a White staff there instead of contacting me who left the contact information and also who make the appointment, but they, for some reason found my partner’s phone number, and then they kind of like contacted directly with him, so I was left out from the diagnosis, or the prescriptions and other information about my cat and that kind of makes me a little disappointed, because I would like to know the first-hand information about my cat.

Lunankin’s experience goes beyond the veterinary team deferring to her White husband in the room when discussing her cat, for whom she is the primary caretaker. Lunankin shares that even after the appointment when she had left her phone number, as her cat’s caretaker, the clinic followed up with her husband.

Mac, who identifies as a Black woman, shared the disrespect she experienced in bringing her dog to the veterinary clinic.

She [the vet] was talking down to me about the food, that she should have had regular hard food…that she should be up to date on these pills, and that her teeth look horrible. She went on and on about all this stuff I had to take her to get a second opinion…it was all of these things that she like was pressuring me and I felt like was making assumptions about who I was. Maybe as a black woman, but in particular as a young black woman, I feel like it was because I was young, and I often have to think like that. I do not think she would have talked to me that way if I were a White man, I just know she would not have. I do not even think she would have had the gall to do it. I even get visibly upset when thinking about that person, that woman, and how I’ve had so many better interactions since. But I just will say that that interaction to me feel very racialized, very gendered, and very targeted at me as a younger black woman with a dog at a vet…I should not have to carry around these assumptions about me. I felt like I was a horrible dog mom after that…I should not have to leave here feeling like a fuck up.

Mac very clearly articulates her experience of intersectionality. She calls out and connects her experience of White paternalistic disrespect to her being not only a Black woman, but a young Black woman, engaging her simultaneous and interconnected experience of racism, sexism, and ageism in her experience with a White, woman veterinarian. She notes how she knows a White man would not have experienced the disrespect and condescension.

Mac also describes the implications for this experience on her personally as well as her trajectory in seeking ongoing veterinary care. Mac describes how her experience with the veterinarian is upsetting even now when she recounts it and how she was made to feel like “a horrible dog mom” and “a fuck up.” Mac also talks about her resulting actions related to veterinary care. She went for a second opinion and did not return to the clinic where she experienced mistreatment as a young Black woman.

Celia, a Latina woman, described the White, paternalistic disrespect she experienced after sharing with her vet that a non-licensed, Chiropractor had treated her dog.

I mean, I think it was in two ways, so, in other words, you know I do not know if he was affected by my gender or if he was just more kind of, you know, very upset at, you know, professionally like his personal beliefs about chiropractors, and his personal beliefs about, you know, the efficacy of me going to take my dog to somebody who’s not licensed. But I think that for me in that interaction, I felt myself kind of like when with that scolding kind of voice like I was caught a little bit off guard and I felt myself kind of falling back into, you know, that space where you back in the day, where you had to be, what’s the word… deferential. To a person of authority, to a man, to a doctor. So it just kind of for me, I felt myself kind of slipping back and not going into my regular kind of mode of just being you know very direct and keeping myself calm and trying to make sure that I’m always, you know, whatever situation it is that I’m the person who’s making the communication happen if there’s not good communication happening.

In her account, Celia names the “deferential” feeling she fell into when being “scolded” by a White, man, Veterinarian. This really illustrates how this theme of disrespect described felt paternalistic for Celia, who felt infantilized in a professional, clinical setting where she was the client.

Many of the participants in recounting their experiences of deference and disrespect also shared how this impacted them by creating self-doubt, where they questioned why they were being mistreated and disrespected and if they were experiencing racism, sexism, and other forms of microaggressions or if it was all in their heads? This is a particularly harmful effect of these racialized and gendered experiences.

Jessica, who identifies as a Latina woman, shared her frustration, self-doubt, and resulting mistrust of her Veterinarian.

I just started to feel like I am no longer believed by my vet. I do not know if it’s because I’m a woman. I do not know if it’s because I’m young. I’m a first-generation American. My family is from Peru, but I do not look it so I can pass as a White person. So, I do not necessarily know ever if they are racially profiling me or if I pass as a White person and it’s something more related to gender, socioeconomic class, or something like that.

The repercussions for many of our participants when experiencing multiple forms of marginalization including racism, sexism, classism, and others included mistrust of their veterinarian, some seeking treatment elsewhere and some tentatively following the veterinary advice received. In one instance, Amber, an African American woman, blamed her veterinarian for the death of her cat when she felt her concerns were dismissed/ignored because she was an African American woman.

## 5 Discussion

These examples are illustrative of the emergent manifestations of intersectionality that we saw in our participants. In their veterinary appointments, some noticed that if there was a White person, especially a White man, in the room with them, the provider tended to direct their communication to them, regardless of who identified as the animal’s primary caretaker in what we referred to as *White/men deference*.

In many instances when communication was directed toward the woman/non-binary client of color, the participants experienced being talked down to and even shamed for a presumed incompetence in caring for their animal, or what we identified as *White paternalistic disrespect*. This experience of being infantilized in their conversations with their veterinary care provider and/or being ignored/rendered invisible stayed with our participants long after their appointments in what we identify as racial/gendered gaslighting. Tyler describes her connections in experience as a child with her White and Black parents, and then her experience with her White husband that linger and cause self-doubt and continued frustration and harm each time she remembers it. Similarly, Mac articulates getting “visibly upset” when she recalls her own treatment at veterinary clinics. Jessica, too, described the impression of feeling doubted by her vet and the lingering effect that has had on her as she wondered which of her identities invoked the doubt.

*Racial/gendered gaslighting* is particularly insidious in the way that it continues to stay with its target long after the interaction, and the way that it can cast self-doubt, i.e., the targeted person wondering if they did something wrong or if it is all in their head. It also haunts those targeted into their next similar experience. For our participants, even though not all of their experiences in veterinary settings have been bad, those where they have had these racial/gendered experiences remain with them as they enter the next veterinary experience, not assuming that they can trust their provider to respect them and/or provide the best care for their animal.

When clients are disrespected and/or their trust in their veterinary team is broken, they are less likely to carry out the recommendations of their veterinary team. They are more likely to seek veterinary care elsewhere, and when there is a dearth of veterinary options and/or the client perpetually has bad experiences in veterinary settings, they are more likely to go without veterinary care for their animals altogether. For women of color, as well as those with other intersectional and marginalized identities, when they experience racism and sexism or other microaggressions from their veterinary team in communication through White paternalistic disrespect or in White, men deferral, they want to remove themselves from those situations to self-protect. It also casts doubt on the medical opinions of the veterinary team, especially in instances where our participants thought they were being offered different options than White men.

Intersectionality, as illustrated by our participants’ narratives, illustrates some of the ways that women/non-binary people of color as clients fall through the cracks of veterinary care in a system that was not designed by nor for them. It is a system that, like Crenshaw noted in the legal system, has rendered multiply marginalized people, like women/non-binary people of color, invisible and not well-served. These dynamics create a barrier in the access to veterinary care for women/non-binary people of color and has borne out in psychological consequences from the mistreatment and continued gaslighting to the perceived subpar care they receive for their animals.

As we have learned from critical race and gender scholars, it is not enough to think of oneself as non-racist or non-sexist, etc. As systems of oppression, such as these, are always at work, so we must always be at work in taking an anti-racist/anti-sexist approach. Intersectional equity-mindedness is one tool toward this. As veterinary professionals design practices, policies, and approaches to care, we must center multiply marginalized communities to identify, prevent, and dismantle the inherent barriers to care we have and continue to build into practices. To have capacity for this, veterinary professionals and the next generation of veterinary professionals, particularly DVM students, must grow their critical consciousness around the experiences of multiply marginalized clients as well as the systems of oppression at work in our society and within clinical settings.

## 6 Conclusion

Author 1 teaches DVM students in social justice issues in veterinary medicine, including unconscious bias and intersectionality. She has noticed that when students start to identify their own unconscious bias, one example that is frequently shared is that when clients are a man and woman couple, they immediately (and often unconsciously) assume that the woman is the caretaker of the animal and they then direct their communication toward her. Author 1 then draws on this research to describe how women of color have shared that when they go to the vet with a white, man partner, the vet tends to talk to the man about the animal care. This has spurred some realization among students that the woman they were assuming was the caretaker was White and allows for a discussion of intersectionality and the unspoken standards that we might push back on as a field using intersectional equity-mindedness.

Within veterinary medicine, trust between the client and provider has an impact not only on the client but on their animal’s health. Trust can determine whether or not the client follows through with the provider recommendations or seeks a second opinion. In areas where there is a dearth of veterinary options, trust can determine whether or not a client seeks veterinary services for their animal in the future. When trust is broken by a client experiencing racism or sexism within veterinary clinical settings, we are reinforcing a barrier to care that jeopardizes the health of those animals.

To engage intersectional equity-mindedness in veterinary medicine, we recommend the following practices:

- Center multiply marginalized communities.
- Create space to consider the needs of multiply marginalized communities.
- Grow critical consciousness among service providers.
- Build support for multiply marginalized clients.
- Dismantle barriers in access to care.

Because our systems, including veterinary medicine, have centered folks with dominant identities, i.e., White, cisgender, men, upper-class, able-bodied, neurotypical, etc., they inevitably fall short for those who do not hold these identities. To then grow access to care, we must center those with marginalized identities in our development of veterinary medical practices, policies, and systems. When we center (or prioritize) multiply-marginalized communities, these systems work for not only those communities but also for everyone.

One way to center multiply-marginalized communities is to create space to consider their needs. In our meetings to discuss policy or reflect on our practices, we can add intersectional equity-mindedness to the agenda. We can make time to ask, ‘Whose needs/access have we not considered in our practice?’ or ‘What possible stakeholders are not at the table that we should connect with in considering this decision?’

When we ask these questions, we sometimes recognize that we do not know the needs and interests of multiply-marginalized communities, and this can serve as an opportunity for our teams to build critical consciousness and to engage in conversations and learning about how systems of oppression might be operating within our processes that prevent access to veterinary care.

Ways to build our critical consciousness include engaging in education connected to social justice and diversity, equity, and inclusion in veterinary medicine. We are fortunate to be seeing a growth in this area within veterinary medicine, including the American Association of Veterinary Medical Colleges (AAVMC) Iverson Bell conferences and the DiVersity Matters Podcast. The American Veterinary Medical Association offered an inaugural DEIW Summit in 2024 and has developed a team-based learning series: Journey for Teams. Beyond this, engaging in fields and literature outside of medicine and continuing to engage with and in research that applies these social justice concepts to veterinary medicine can build our collective critical consciousness, along with our individual ones.

In doing this work, we will identify places where we can build supports to promote access to veterinary care. For instance, are our services accessible for those who do not, or prefer not to speak English in clinical settings? Are our clinic hours accessible for those who cannot take off work during business hours or have caretaking responsibilities for children or elders? More relevant to this study, have we and our teams engaged in education and team-building to ensure we can show respect and empathy across cultures and identities?

Some barriers to access can more immediately be addressed through supportive measures, and others are more rooted in the systems and cannot be immediately addressed. However, when we are aware of these barriers, we are then able to look for opportunities over time where we can dismantle them. For instance, if our buildings are not accessible for disabled people or we do not have all-gender restrooms available, we may not have funds to immediately remedy these barriers, but when we know they are there, we can be ready to address them in the future when we make renovations or build future structures.

Because the field of veterinary medicine remains overwhelmingly White, we need to meaningfully engage intersectional equity-mindedness into our practices and into the foundational education and continuing education of providers. We need to expand our understanding of access to care to include the dismantling of social barriers that remain within veterinary medicine as a system. At the same time, we must continue to expand access to the field to ensure that folks with multiple marginalized identities can see themselves in the field.

## Data Availability

The datasets presented in this article are not readily available because the data consists of interview transcripts obtained with informed consent and not shareable by those who are not IRB-approved investigators on this project. Requests to access the datasets should be directed to naomi.nishi@colostate.edu.

## Footnotes

^1^We define access to veterinary care as the removal of physical, socioeconomic, social, emotional barriers that inhibit a person’s ability to seek quality healthcare for their animal (4).

^2^We use “multiply” as an adverb rather than a verb in alignment with Patricia Hill Collins’ concept of being multiply marginalized (14).
